# Toxicity Evaluation and Transcriptome Analysis of Yellowstripe Goby (*Mugilogobius chulae*) in Response to 2,7-Dibromocarbazole Exposure during Early Development

**DOI:** 10.3390/toxics12080609

**Published:** 2024-08-20

**Authors:** Caixia Gao, Suqun Lai, Jin Zeng, Ying Peng, Jianjun Li

**Affiliations:** 1 Guangdong Provincial Biotechnology Research Institute (Guangdong Provincial Laboratory Animals Monitoring Center), Guangzhou 510663, China; caic2665@163.com (C.G.); 13751742796@163.com (S.L.); 15807538569@163.com (J.Z.); 2 Research and Development Center for Watershed Environmental Eco-Engineering, Advanced Institute of Natural Sciences, Beijing Normal University, Zhuhai 519087, China; pengying@bnu.edu.cn; 3 Key Laboratory of Coastal Water Environmental Management and Water Ecological Restoration of Guangdong Higher Education Institutes, Beijing Normal University, Zhuhai 519087, China; 4 School of Environment, Beijing Normal University, Beijing 100875, China

**Keywords:** *Mugilogobius chulae*, 2,7-DBCZ, fish embryo acute toxicity (FET), concentration–effect curves, differentially expressed genes (DEGs)

## Abstract

Polyhalogenated carbazoles (PHCZs) are a class of nitrogen-containing heterocyclic compounds that are widely distributed throughout the marine environment and sediment. These compounds share structural and toxicity similarities with dioxins. However, our understanding of the toxicological effects of PHCZs on marine organisms and their underlying molecular mechanisms remains limited. In this study, we employed the marine model organism *Mugilogobius chulae* as the experimental subject and selected 2,7-dibromocarbazole (2,7-DBCZ), a compound known for its high toxicity and detection frequency, to conduct both an acute toxicity test and transcriptome analysis on *M. chulae* embryos. Our findings revealed that the 96 h median lethal concentration (LC_50_) of 2,7-DBCZ for *M. chulae* embryos was 174 μg/L, with a median effective concentration (EC_50_) resulting in pericardial edema deformity of 88.82 μg/L. Transcriptome analysis revealed significant impacts on various systems in *M. chulae* embryos following exposure to 2,7-DBCZ, including the sensory, cardiovascular, immune, and endocrine systems. Furthermore, this compound perturbed signaling pathways such as phototransduction, protein folding and processing, amino acid metabolism, lipid transport, and exogenous compound metabolism. Notably, transcript abundance of the *CYP1A* gene associated with the activation of the AhR signaling pathway, similar to dioxin-like compounds, was 18.18 times higher than that in the control group. This observation suggests that *M. chulae* embryos mount a stress response when exposed to PHCZs. In summary, this study contributes to our understanding of the toxicological implications of PHCZ in marine fish and offers a theoretical foundation for risk assessment and regulatory frameworks for PHCZs in the marine environment.

## 1. Introduction

Carbazoles and their derivatives are a class of nitrogen-containing heterocyclic compounds with a wide range of applications. Polyhalogenated carbazoles (PHCZs) are a subgroup of compounds in which hydrogen atoms on the carbazole ring are replaced by halogen atoms (C1, Br, or I). These compounds share structural similarities with dioxins and have been detected in both the environment and in living organisms [1]. Due to their high detection rates and dioxin-like toxic effects, they have garnered increasing attention in recent years. There are two primary sources of PHCZs in the environment: natural occurrences and anthropogenic emissions. Through enzymes such as chloroperoxidase and carbazole, marine fungi are capable of synthesizing BCZs and CCZs via enzymatic reactions. Volcanic eruptions and forest fires can also generate PHCZs [2]. The anthropogenic sources of PHCZs include dye production [3], intermediates used in the synthesis of optoelectronic materials, pharmaceutical manufacturing, and herbicide usage [4]. Furthermore, the chlorination and disinfection of drinking water under specific conditions may lead to the formation of PHCZs [5].

PHCZs are widely distributed in nature and have been detected in sediment, soil, air, water, and biological samples. Their concentrations vary significantly across different environmental media [6]. In China, PHCZ residues in sediments have been primarily found in Taihu Lake, the Jiulong River in Fujian Province, Sanmen Bay in the East China Sea, the Jiaozhou Bay wetland, and the intertidal zone of the New River Estuary. The detected concentrations of PHCZs ranged from 0.34 to 61.15 ng/g [7]. PHCZs in soil were detected around tie-dye workshops in Yunnan, with concentrations ranging from 1.5 to 14.6 pg/g [8]. Additionally, PHCZs have been detected in water samples from the East China Sea, the Yellow Sea, and Wuhan, with concentrations ranging from 0.062 to 53.48 ng/L [9,10,11]. PHCZs have also been found in various aquatic and marine organisms, including shells, fish, seals, and cormorant eggs, at concentrations ranging from 33.7 to 164 ng/g [12]. Given the resilience of PHCZs in the environment, the associated environmental and health risks cannot be overlooked.

Among all the PHCZs detected in the environment, BCZs and CCZs are the two most prevalent, with BCZs exhibiting the highest degree of toxicity. This group includes compounds such as 3,6-DBCZ and 2,7-DBCZ. Ji et al. [13] demonstrated that 2,7-DBCZ had the highest toxicity to zebrafish, with a 96 h LC_50_ value of 581.8 ± 29.3 μg/L. Exposure to 2,7-DBCZ led to significant pericardial edema and a marked upregulation of the aromatic hydrocarbon receptor (AHR) in zebrafish. Du et al. [6] also reported that 3,6-DBCZ induced a range of toxic effects in zebrafish, including pericardial edema, short tails, and spinal curvature, with a 96 h LC_50_ value of 1.167 mg/L. Moreover, 3,6-DBCZ exposure treatment leads to a significant reduction in axon length and number in zebrafish motoneurons [14]. In contrast, combined exposure to 3,6-DBCZ and PS-MPs significantly reduced the levels of oxidative stress and apoptosis in zebrafish embryos [15]. Moreover, both 3,6-CCZ and 2,7-BCZ have high bioaccumulation potential in aquatic systems [16]. These studies effectively illustrate the potential toxic effects of BCZs on freshwater fish. In marine organisms, exposure to 3,6-CCZ was significantly correlated with the ratio of ∑n − 6/∑n − 3 polyunsaturated fatty acids (PUFA) in harbor seals, and these contaminants may disrupt lipid metabolism in marine mammals [17]. However, additional studies are still needed to elucidate the biotoxic effects of BCZs on marine fish.

*Mugilogobius chulae,* a member of the Perciformes order within the Gobiidae family, is a small warm-water marine fish species. They are widely distributed in the South China Sea, East China Sea, and other coastal waters of the western Pacific Ocean, and they are the only seawater experimental fish for which national quality control standards have been established in China [18]. *M. chulae* possesses a variety of advantageous attributes such as small size, high reproductive capacity, transparent embryos, a short reproductive cycle, and ease of captive breeding [19]. Due to these advantages, *M. chulae* embryos are considered an ideal experimental model for ecotoxicological studies in marine environments, and early larvae are widely utilized in ecotoxicological investigations involving heavy metal pollutants [20], organic compounds [21], inorganic substances [22], pharmaceuticals [23], and drilling fluids [24], among others.

Nevertheless, there is still a lack of research concerning the evaluation of the toxicity of PHCZs in marine fish. Therefore, this study sought to assess the toxic effects of 2,7-DBCZ, one of the most potent PHCZs, on *M. chulae* embryos. The assessment involved a 96 h acute toxicity test and transcriptome sequencing analysis to elucidate the mechanism of toxicity of 2,7-DBCZ in *M. chulae*. This research provides novel insights into the toxicity of PHCZs, particularly 2,7-DBCZ, in marine fish and may serve as a basis for the development of detection and control strategies for PHCZs in marine environments.

## 2. Materials and Methods

### 2.1. Ethical Statement

All experiments involving the *M. Chulae* have been reviewed and approved by the Institutional Animal Care and Use Committee (IACUC) of Guangdong Laboratory Animal Monitoring Institute (project certificate No. IACUC2024129). The health of the animals was monitored daily during the experiment.

### 2.2. Acute Embryotoxicity of 2,7-DBCZ

The PHCZ, 2,7-dibromocarbazole (2,7-DBCZ, CAS#: 136630-39-2), (98% purity) was procured from Shanghai Macklin Blochemical Co., Ltd. (Shanghai, China). Dimethyl sulfoxide (DMSO, CAS#: 67 68-5) was obtained from Sangong Biotech (Shanghai) Co., Ltd. (Shanghai, China). Stock solutions of the chemical were prepared in DMSO, at a 2 g/L concentration. The stock solutions were then further diluted to achieve the required test concentrations. The working solution of *M. chulae* embryos was prepared using filtered and sterilized natural seawater.

### 2.3. Cultivation and Sampling of M. chulae Embryos

The *M. chulae* parents were bred and raised in our laboratory. Male parents had an average total length of 32.15 ± 3.12 mm and a body weight of 346.23 ± 4.83 mg, while female parents had an average total length of 29.33 ± 4.56 mm and a body weight of 254.92 ± 7.05 mg. Sexually mature male and female parents were paired in a 1:1 ratio and placed in a breeding tank with a volume of 3 L (20 × 10 × 15 cm). The water used for breeding was sand-filtered seawater, and the breeding was conducted in a recirculation aquaculture system. Seawater temperature, salinity, and dissolved oxygen were maintained at 25 ± 1 °C, 20‰, and 7.01 ± 0.75 mg/L, respectively, with a 4 h light and 10 h dark photoperiod. *M. chulae* were fed twice daily with artificial forage. To facilitate the breeding process, nests were placed in the tank in advance. Once both the male and female parents simultaneously entered the nest, they engaged in natural mating, a process that typically lasted 1–2 h. After spawning, the nests were removed and the fertilized eggs were gently extracted using forceps and then transferred to clean containers with an *M. chulae* embryo working solution for subsequent experiments.

### 2.4. Developmental Toxicity of M. chulae Embryos

The fish embryo acute toxicity (FET) test was optimized according to the OECD 236 guidelines [25]. Exposure concentrations of 2,7-DBCZ were set at 250, 200, 150, 100, and 50 μg/L. A solvent control group (containing embryo working solution with 0.1% DMSO) and a blank control group were also included in all experiments. The FET test was performed in a 24-well plate, with each well containing 2 mL of the exposure solution and one embryo at 2 h post-fertilization (hpf). Each concentration group consisted of a total of 24 embryos, with each experiment being conducted in triplicate. After transferring the embryos to the wells, the 24-well plates were placed in a thermostatic incubator set at 25 ± 1 °C with a 14 h light and 10 h dark cycle. To maintain the accuracy of the exposure concentrations, half of the exposure solution was replaced every 24 h during the test. The embryos were observed every 24 h, with developmental indicators including heart, yolk sac, body axis, tail, lower jaw, fin, swim bladder, eyes, brain development, and hatching being monitored. After the exposure, the mortality rate and malformation rate of each group were tallied and concentration–effect curves were generated.

### 2.5. Sample Preparation for Transcriptome Sequencing

Based on the results obtained from the FET test, an exposure concentration of 100 μg/L for 2,7-DBCZ was selected for transcriptome sequencing. An embryo working solution containing 0.1% DMSO was established as the solvent control group. The experiments were conducted in 6-well plates, with each containing 10 mL of exposure solution and twenty 2 hpf embryos. Each group was analyzed in triplicate (120 embryos per replication), for a total of 360 embryos in each group. After 96 h of exposure, all embryos from both the 100 μg/L exposure group and the solvent control group were collected, and the samples were subjected to three washes with phosphate-buffered saline (PBS), gently blotted dry, and then transferred to sterilized centrifuge tubes. These tubes were rapidly frozen in liquid nitrogen, and the samples were subsequently stored in a −80 °C refrigerator.

### 2.6. RNA Extraction, Library Preparation, and Sequencing

Transcriptome sequencing was conducted by Majorbio Bio-pharm Biotechnology Co., Ltd. (Shanghai, China). Total RNA was extracted from the embryo samples using the TRIzol^®^ Reagent (Invitrogen, Carlsbad, CA, USA) according to the manufacturer’s instructions. The concentration and purity of the isolated RNA were assessed using an ND-2000 spectrophotometer (NanoDrop Technologies, Wilmington, DE, USA), and the RIN (RNA Integrity Number) value was determined using an Agilent 5300 Fragment Analyzer. The RNA-seq transcriptome library of the *M. chulae* embryos was prepared using the Illumina^®^ Stranded mRNA Prep Ligation Kit from Illumina (San Diego, CA, USA) and 1 μg of total RNA. Double-stranded cDNA was synthesized using the SuperScript double-stranded cDNA synthesis kit (Invitrogen, CA, USA) with random hexamer primers from Illumina. Subsequently, the synthesized cDNA underwent end-repair, phosphorylation, and ‘A’ base addition following Illumina’s library construction protocol. The paired-end RNA-seq sequencing library was sequenced on a NovaSeq 6000 sequencer (2 × 150 bp read length) (Illumina, San Diego, CA, USA). Raw paired-end reads were trimmed using Fastp [26], after which the clean reads were aligned to the reference genome using the HISAT2 software [27]. The mapped reads for each sample were then assembled using StringTie [28].

### 2.7. Differential Expression Analysis and Functional Enrichment

To identify differentially expressed genes (DEGs) between control and exposed samples, the expression level of each transcript was calculated in terms of transcripts per million (TPM) reads. Gene abundance was quantified utilizing RSEM [29]. The analysis of differential gene expression was carried out using either DESeq2 [30] or DEGseq [31]. DEGs meeting the criteria of |log2FC| ≥ 1 and FDR ≤ 0.05 (DESeq2) were considered to be significantly differentially expressed genes. Functional enrichment analyses including GO and KEGG were also conducted to gain insights into the biological functions of the identified DEGs. GO enrichment analysis was performed using the GOatools software (https://github.com/tanghaibao/GOatools, accessed on 29 May 2023) and statistical significance was evaluated using Fisher’s exact test. To control for the calculated false-positive rate, the p-values were corrected using the Bonferroni, Holm–Sidak, and false discovery rate tests. GO gene pathways were considered significantly enriched when the corrected *p*-value ≤ 0.05. KEGG pathway enrichment analysis was performed using KOBAS (https://bio.tools/kobas, accessed on 29 May 2023), and calculations were performed using Fisher’s exact test. To control for the calculation of false-positive rates, multiple testing was performed using the Bonferroni and false discovery rate methods, with a corrected *p*-value threshold of 0.05. KEGG pathways meeting this condition were considered to exhibit significant Deg enrichment [32].

### 2.8. Protein-Protein Interaction Networks Analysis

To investigate the protein–protein interaction (PPI) networks among all the DEGs identified in the KEGG-enriched pathways, a network modeling approach was employed. This analysis involved examining protein interactions online using the String database (https://string-db.org/, accessed on 8 August 2023). The aim was to uncover and explore the interaction relationships among important proteins based on the transcriptome results.

### 2.9. Quantitative Real-Time PCR (qPCR) Validation

qPCR analyses were conducted to validate the RNA-seq results. For the design of primers, 10 DEGs (comprising 6 up-regulated and 4 down-regulated genes) were selected based on their functional attributes, genomic location, and expression levels (as detailed in Table 1). *β-Actin* served as the reference gene, and the primers were synthesized by Sangon Biotech. Quantitative polymerase chain reaction (qPCR) amplification was conducted using a QuantStudio 5 instrument (Applied Biosystems, Thermo Fisher Scientific, Waltham, MA, USA). All reactions were conducted using SYBR Premix Ex Taq ^TM^ II (Takara, Dalian, China) and the reaction program consisted of an initial denaturation step at 95 °C for 30 s, followed by 40 cycles of denaturation at 95 °C for 5 s, and annealing/extension at 60 °C for 43 s. Relative mRNA expression was calculated using the 2^−ΔΔCt^ method [33].

### 2.10. Statistical Analyses

Each experiment was independently conducted in triplicate. All experimental data are presented as the mean ± standard deviation (SD) and analyzed with the SPSS 26 software (Chicago, IL, USA). The normality of distributions and homogeneity of variances were tested using the Kolmogorov–Smirnov test and Levene’s test, respectively. Non-parametric analyses were conducted using the Mann–Whitney U test and the Kruskal–Wallis for pair-wise and multiple comparisons, respectively. A *p*-value < 0.05 was considered statistically significant. Graphs were generated using GraphPad 7.01 (San Diego, CA, USA).

## 3. Results

### 3.1. Acute Embryo Toxicity of 2,7-DBCZ

**Figure 1:**
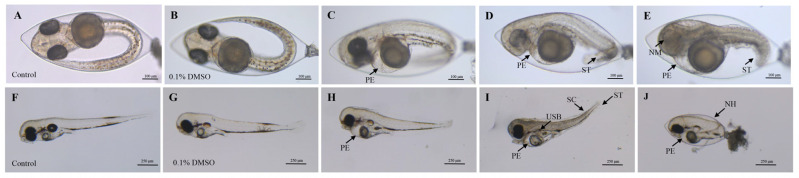
Deformities of *M. chulae* embryos resulting from 2,7-DBCZ exposure. (**A**–**E**): Embryos exposed for 48 h ((**A**): Blank control group, (**B**): Solvent control (0.1% DMSO), (**C**–**E**): 2,7-DBCZ groups). (**F**–**J**): Embryos exposed for 96 h ((**F**): Blank control group, (**G**): Solvent control (0.1% DMSO), (**H**–**J**): 2,7-DBCZ groups). PE: pericardial edema; ST: short tail; NM: no melanin in the lens; SC: spinal curvature; USB: uninflated swim bladder; NH: non-hatching.

The 96 h FET test revealed varying toxic effects of 2,7-DBCZ on *M. chulae* embryos at different concentrations. Initially, all test and exposure groups of *M. chulae* embryos exhibited normal development from the start of exposure up to 24 h. At 48 h, embryos in all groups exhibited heartbeat activity. Compared to the blank control group (Figure 1A), the embryos in the solvent control group developed normally (Figure 1B), whereas the 250 and 200 μg/L exposure groups displayed evident developmental retardation. One of the most notable features of the affected embryos included the absence of melanin deposits in the eye lens, along with morphological deformities such as short tails, spinal curvature, and underdeveloped pericardial chambers, in addition to the occurrence of embryonic tremors. Pericardial edema was observed in the groups exposed to 150 and 100 μg/L (Figure 1C–E).

**Figure 2:**
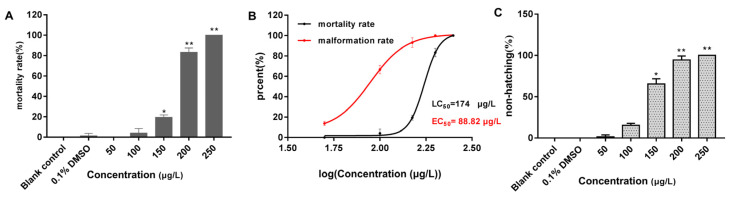
Mortality, deformity, and non-hatched rate of *M. chulae* embryos after 2,7-DBCZ exposure for 96 h. (**A**) Histogram of embryonic mortality in *M. chulae* embryos, data are presented as mean ± SD (n = 3), *p* < 0.05 (*); *p* < 0.01 (**); (**B**) Concentration–effect curves in the development of *M. chulae* embryos; (**C**) Histogram of embryonic non-hatching in *M. chulae* embryos, data are shown as mean ± SD (n = 3); *p* < 0.05 (*), *p* < 0.01 (**).

All embryos in the 250 μg/L group had perished by the 96 h mark. Compared with the blank control group (Figure 1F), the embryos in both the solvent control (0.1% DMSO) (Figure 1G) and 50 μg/L groups developed normally, whereas the other exposure groups still exhibited developmental abnormalities, most notably pericardial edema (Figure 1H–J). As shown in Figure 2, the mortality rate of the solvent control group was 1.39%, whereas the mortality rates in the exposure groups ranged from 0 to 100%, with mortality increasing in a concentration-dependent manner (Figure 2A). The 96 h LC_50_ value of 2,7-DBCZ for *M. chulae* embryos was calculated to be 174 μg/L based on mortality in each group (Figure 2B). Additionally, the median effective concentration (EC_50_), resulting in pericardial edema deformity in *M. chulae* embryos, was determined to be 88.82 μg/L (n = 24 samples) based on pericardial edema calculations (Figure 2B). Additionally, the non-hatching rate of *M. chulae* embryos in the blank and solvent control groups was 0, the non-hatching rate of each concentration group after 2,7-DBCZ exposure increased gradually with the increase of concentrations, which were 1.39, 15.28, 65.28, 94.45 and 100%, respectively (Figure 2C).

### 3.2. Overview of Transcriptional Analysis

Transcriptome analyses were conducted on a total of six samples from the 2,7-DBCZ group and the control group and the percentage of Q30 bases exceeded 94.33% (Table 2). Sequence comparison was performed separately for the clean reads of each sample against the designated reference genome. The comparison rates for total mapped, multiple mapped, and uniquely mapped reads ranged from 82.71 to 84.64%, 3.66 to 4.30%, and 78.84 to 80.38%, respectively (Table 3).

**Figure 3:**
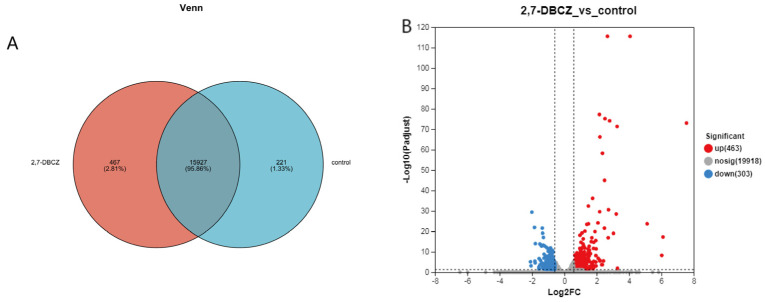
Results of DEGs in the embryos after 2,7-DBCZ exposure. (**A**) Venn diagram between samples. Circles of different colors represent genes in a group screened based on expression, and the values represent the number of genes shared and unique among different samples. (**B**) Volcano plot of differentially expressed genes (DEGs). Up-regulated differential genes are labeled with red dots, down-regulated differential genes are labeled with blue dots, and non-differential genes are labeled with gray dots in panel.

In this analysis, a total of 34,359 expressed genes were detected, comprising 20,684 known genes and 13,675 new genes. Additionally, 68,220 expressed transcripts were detected, including 34,693 known transcripts and 33,527 new transcripts. Furthermore, 221 genes were specifically expressed in the control group, and 467 genes were specifically expressed in the 2,7-DBCZ-exposed group (Figure 3A). A DEGs analysis was performed between the two groups using the DESeq2 software (|log2FC| ≥ 0.585 and P_adjust_ < 0.05). A total of 766 DEGs were identified in the 2,7-DBCZ group compared to the control group, comprising 463 significantly up-regulated genes and 303 significantly down-regulated genes (Figure 3B).

### 3.3. GO Functional Analysis of DEGs

**Figure 4:**
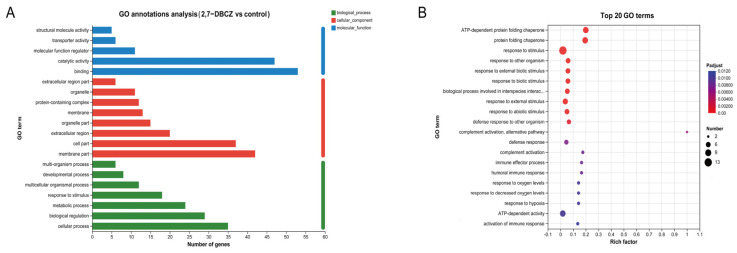
GO analysis of DEGs. (**A**) Column chart of GO classification statistics. The vertical coordinates indicate secondary classification terms of GO, the horizontal coordinates indicate the number of genes corresponding to that secondary classification, and the three colors indicate the three major classifications. (**B**) Top 20 enriched GO terms. The size of the dots indicates the number of genes in this GO Term, and the color of the dots corresponds to different P_adjust_ ranges.

GO functional annotation was conducted to classify the functions of all DEGs into three main categories, as illustrated in Figure 4A. All DEGs were enriched in a total of 186 pathways, mainly distributed in the cellular process of the biological process, the membrane part of the cellular component, and the binding of the molecular function. Among these, 125 pathways were enriched in biological processes. The significantly enriched pathways included complement activation, immune effector processes, humoral immune response, response to stimuli, and activation of immune response (P_adjust_ < 0.05). Furthermore, 46 pathways were enriched in the molecular function category, with significantly enriched pathways such as the protein folding chaperone and ATP-dependent protein folding chaperone. Additionally, 15 pathways were enriched in the cellular component category, with significant enrichment observed exclusively in the extracellular region pathway (Figure 4B). The GO functional enrichment analysis indicated that exposure to 2,7-DBCZ had a more pronounced impact on biological processes, interfered with intracellular protein folding processes, and triggered a robust immune response in the organism.

### 3.4. KEGG Pathway Analysis of DEGs

**Figure 5:**
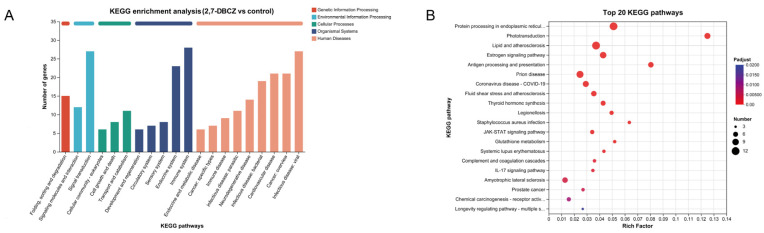
KEGG analysis of DEGs. (**A**) Bar chart of KEGG classification statistics. The horizontal coordinate indicates the names of the KEGG metabolic pathway; the vertical coordinate is the number of genes or transcripts annotated to the pathway. (**B**) Top 20 enriched KEGG signaling pathways. The size of the dots indicates the number of genes in this KEGG term, and the color of the dots corresponds to different P_adjust_ ranges.

Utilizing KEGG enrichment analysis, the identified DEGs were further annotated with standardized gene functions and meanings, as shown in Figure 5A. All DEGs were enriched in a total of 219 KEGG pathways. These pathways were mainly involved in the folding, sorting, and degradation of genetic information processing; the signal transduction of environmental information processing; the transport and catabolism of cellular processes; the immune system organismal systems; and infectious disease, specifically viral infections. Of these pathways, 12 were significantly enriched (P_adjust_ < 0.05). The top five enriched pathways included phototransduction, protein processing in the endoplasmic reticulum, lipids and atherosclerosis, antigen processing and presentation, and the estrogen signaling pathway. Additionally, the fluid shear stress and atherosclerosis pathways, associated with lipid metabolism, exhibited significant enrichment. It is also worth noting that among the top 20 KEGG enriched pathways (Figure 5B), the drug metabolism–cytochrome P450 signaling pathway and the JAK-STAT signaling pathway, both linked to the metabolism of exogenous compounds, were also enriched. Furthermore, the results of our KEGG enrichment analyses revealed that exposure to 2,7-DBCZ affected the sensory, cardiovascular, immune, and endocrine systems, as well as genetic information processing and amino acid metabolism in *M. chulae* embryos. It also interfered with visual formation and development and amino acid transport and catabolic processes.

### 3.5. PPI Analysis

**Figure 6:**
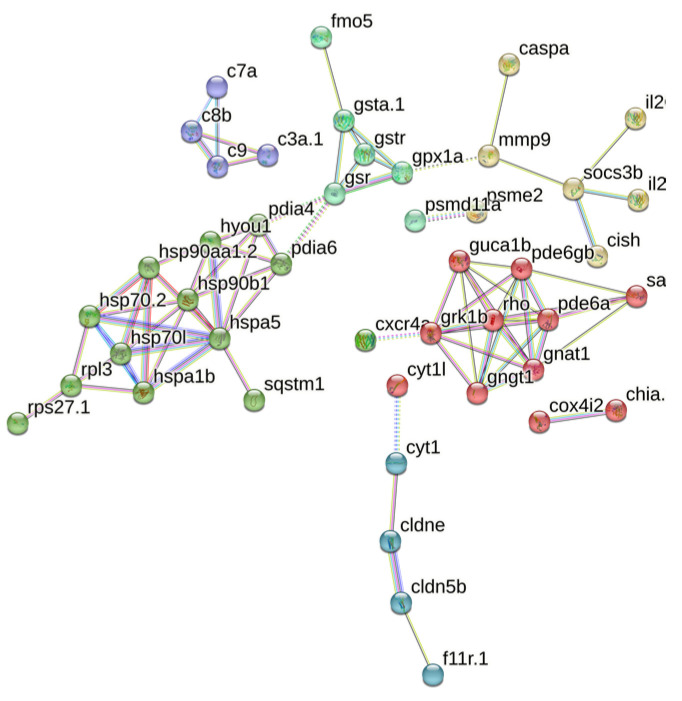
PPI analysis of core genes. The network nodes represent proteins, the links between the nodes represent protein–protein interactions, and the colors represent six clusters.

A total of 95 matches were retrieved from the String database, with 45 proteins interacting with each other. These 45 proteins were subjected to cluster analysis using the k-means clustering method, resulting in six groups of proteins with distinct functions (Figure 6). Among these, the 20 most important hub proteins were identified, including hspa5, hsp90b1, hyou1, gsr, gpx1a, rpl3, pde6gb, pde6a, gngt1, gnat1, pde6a, pdia6, rho, grk1b, gngt1, c8b, c9, c3a.1, socs3b, and cldne. These hub proteins are primarily associated with four clusters centered on phototransduction, metabolism of exogenous compounds, folding and processing of endoplasmic reticulum proteins, and the complement system. For instance, fmo5, gsta1, gstr, and gsr collectively mediate biological oxidation, whereas hspa5, hsp90b1, hspa1b, and hsp70.2 are involved in the regulation of protein folding and processing in the endoplasmic reticulum. Additionally, c3a.1, c7a, c8b, and c9 are part of the innate immune system. These proteins not only exhibit close intra-group relationships but also display complex interactions between different groups.

### 3.6. qPCR Verification of Transcriptional Analyses

**Figure 7:**
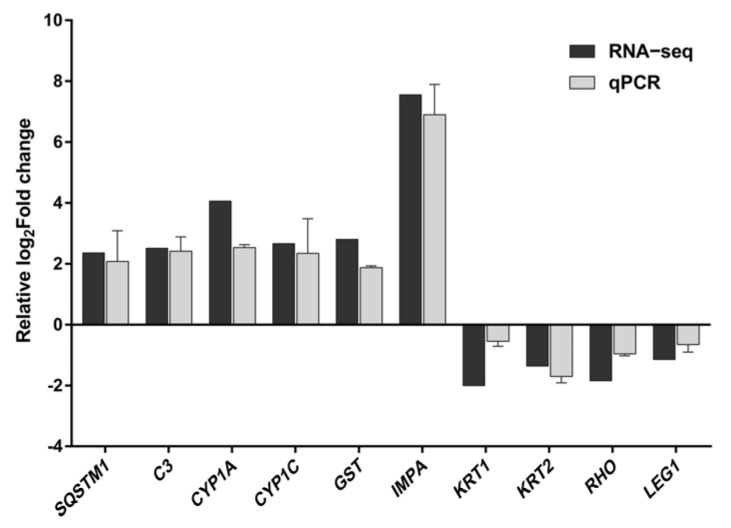
Comparison of the relative log2Fold change between RNA-Seq results and qPCR. Each value represents the mean ± SD of three independently replicated tests. The RNA−Seq values represent the average of three replicates.

The results of transcriptome sequencing were validated through qPCR assays, demonstrating that the expression levels of mRNA for 10 representative genes were consistent with the findings from the transcriptome sequencing (Figure 7).

## 4. Discussion

With the advancement of detection technology and the growing concern over environmental pollutants, the detection of PHCZs has become increasingly more frequent. In this study, we selected the marine model organism *M. chulae* to assess 2,7-DBCZ toxicity. The results from the FET test showed that the 96 h LC_50_ of 2,7-DBCZ for *M. chulae* embryos was 174 μg/L, categorizing it as highly toxic (<1 mg/L). In contrast, a study using zebrafish as a model organism reported a 96 h LC_50_ and EC_50_ for 2,7-DBCZ of 581.8 and 201.5 μg/L, respectively [13], which were 3.3-fold and 2.27-fold higher than the toxicity observed in *M. chulae* embryos. This disparity suggests that different organisms exhibit varying levels of toxicity response to 2,7-DBCZ and underscores the sensitivity of *M. chulae* embryos to the toxicity of 2,7-DBCZ, a conclusion supported by the EC_50_ and LC_50_ results. Therefore, a new evaluation system is needed for assessing the effects of 2,7-DBCZ on marine fish relative to freshwater organisms. Similarly, the large difference in the LC50 of TCDD between freshwater and marine organisms supports our inference. In conclusion, these findings highlight the importance of our proposed toxicity evaluation system for 2,7-DBCZ in marine organisms [34,35].

In all PHCZ toxicity studies, the most typical toxic effect in fish was pericardial edema, which was often accompanied by several other dysmorphic phenotypes, such as the absence of melanin deposits in the eye lens, spinal curvature, short tail, and transparent body surface [6,36]. In our study, the toxic responses of *M. chulae* embryos to 2,7-DBCZ varied at different concentrations. Significant developmental retardation and embryo death before hatching occurred in the higher concentration groups (250 and 200 μg/L). In contrast, the most prevalent malformation in embryonic development in groups below 150 μg/L was pericardial edema, with an EC_50_ of 88.82 μg/L. Additionally, the 150 μg/L group exhibited an absence of melanin deposits in the eye lens, spinal curvature, short tails, and other malformations, which was consistent with earlier findings in zebrafish [13]. Previous research has shown that the area of pericardial edema in zebrafish is positively correlated with 2,7-DBCZ concentration. Ji et al. [13] investigated 2,7-DBCZ-induced pericardial edema using transgenic zebrafish and found that treatment with 0.5, 1, and 2 μmol/L 2,7-DBCZ led to 1.68-, 2.09-, and 21.81-fold increases in pericardial edema area, with an EC_50_ value of 201.5 μg/L. Fang et al. [36] also demonstrated a significant dose-dependence of pericardial edema in zebrafish, attributing it to the expression of the CYP1A gene and protein synthesis, consistent with observations of 2,3,7,8-TCDD. Du et al. [6] discovered that in addition to pericardial edema, which is a common outcome of PHCZ exposure in zebrafish, high concentrations of 3,6-DCCZ and 3,6-DBCZ hindered heart rate and increased SV-BA distance. Interestingly, *Xenopus tropicalis*, a model amphibian species, also exhibited pronounced pericardial edema when exposed to 2,7-DBCZ, accompanied by a reduced heart rate. Moreover, 2,7-DBCZ induced mitochondrial swelling in *X. tropicalis* embryo cardiomyocytes, leading to cardiac dysfunction [37]. Collectively, these studies highlight cardiotoxicity as one of the prominent toxic effects of 2,7-DBCZ, observed in *X. tropicalis*, zebrafish, and *M. chulae*.

Transcriptome sequencing serves as a valuable tool in biotoxicity studies, offering insights into the molecular mechanisms within organisms in response to toxicant exposure. In our study, we employed transcriptome analysis to explore molecular-level alterations in *M. chulae* embryos following exposure to 100 μg/L of 2,7-DBCZ. This analysis aimed to identify major biochemical processes and metabolic pathways affected by the DEGs and to provide an initial understanding of the toxic mechanisms involved. Compared to the control group, we identified 766 DEGs in goby embryos exposed to 2,7-DBCZ. Remarkably, this number significantly exceeded the 90 DEGs reported in zebrafish embryos exposed to 0.6 μmol/L of 2,7-DBCZ in previous studies [13], whereas 100 μg/L 2,7-DBCZ exposure resulted in 840 DEGs in tropical *Xenopus laevis* embryos [37]. These variations could be attributed to species-specific differences or disparities in the concentration and duration of exposure. Notably, our findings suggest that *M. chulae* embryos exhibit higher sensitivity compared to zebrafish embryos, aligning with the LC_50_ results.

Remarkably, genes associated with pericardial edema were significantly differentially expressed in these 766 DEGs, including *CYP1A*, *CYP1B1*, and *AhR*. Cytochrome P450s (CYPs) represent a crucial family of enzymes responsible for catalyzing the oxidation and biotransformation of a wide range of drugs and other lipophilic exogenous compounds [38]. Among them, *CYP1A* serves as a key regulator of exogenous compound metabolism, particularly in the oxidation and conversion of planar aromatic hydrocarbons [39]. When dioxin-like compounds activate the AhR signaling pathway, the expression of the *CYP1A* gene significantly increases [40]. Therefore, the expression level of *CYP1A* serves as a typical biomarker for AhR signaling pathway activation. In our study, the upregulation of *CYP1A1* was observed to be 18.18 times higher than that in the control group, suggesting a robust response by *M. chulae* embryos to the presence of exogenous compounds. Furthermore, the expression of *CYP1C* exhibited a significant up-regulation, reaching a 6.87-fold difference compared to the control. A previous study demonstrated a positive correlation between the expression of genes like *CYP1A* and *CYP1B1*, associated with AhR pathway activation, and the dose in zebrafish embryos following dioxin exposure. This resulted in pericardial edema, tissue edema, or even embryo mortality [41]. In contrast, similar toxic effects and molecular mechanisms are present in the *M. chulae* embryos. We hypothesize that the cardiac teratogenicity induced by 2,7-DBCZ in *M. chulae* embryos may be linked to the AhR-mediated signaling pathway.

Furthermore, GO functional enrichment analysis revealed a significant enrichment of GO terms related to protein folding, complement activation, and immune response. Protein folding plays an essential role in the development of the embryo and the nervous system [42]. Disruption of protein folding causes neurological disorders and embryonic mortality [43,44]. Based on these observations, our findings suggest that 2,7-DBCZ exposure affects the protein folding pathways, thereby impairing eye and spine development and increasing mortality rates in *M. chulae*. The complement system promotes the polarization and proliferation of embryonic neural progenitor cells [45]. Moreover, the complement system is not only an important component of innate immunity [46] but is also critical for embryonic development and lipotrophic homeostasis [47]; C3, in particular, serves as a central effector molecule within the complement system, mediating multiple functions through different binding sites and their corresponding receptors [48]. Both C8 and C9 function as components of the soluble membrane attack complex, which can insert into the lipid bilayer, disrupting cellular integrity and function [49]. Additionally, certain complement components are known to play crucial roles in the development of the central nervous and visual systems [50,51]. This is similar to previously reported observations in *X. tropicalis* toads after 2,7-DBCZ exposure [37], further suggesting that 2,7-DBCZ affects *M. chulae* embryonic development and damages the optic and vertebral nerves by impairing the homeostasis of the complement system. Treatment with 2,7-DBCZ and its analogs induces oxidative stress in embryos [6,13], and activation of the immune response pathway is an important means for organisms to respond to the repair of oxidative stress damages caused by exogenous stimuli [13,52].

Functional enrichment pathway analysis revealed a significant enrichment of KEGG pathways related to phototransduction, lipid metabolism, and cardiovascular development. The phototransduction pathway, an important link in biological visual development, is essential for visual imaging and eye development [53,54]. Specifically, the *Rho*, *Gngt1*, *Pde6a*, *Pde6d*, *Grk1*, and *Sag* genes, which are associated with this pathway, displayed significant down-regulation. Rhodopsin (Rh) is a member of the extensive G-protein-coupled receptor family [55] and plays a key role in phototransduction by activating Gt proteins that transmit light signals to the nervous system [56]. Given that 2,7-DBCZ exposure induced the phototransduction pathway, it may affect the normal development of goby eyes. Simultaneously, we observed significant enrichment in the lipid and atherosclerosis signaling pathway, coinciding with a substantial suppression of apolipoprotein *A-IV* (*apoA-IV*) expression. A-IV protein is involved in a multitude of physiological processes, including lipid uptake and metabolism, protection against atherosclerosis, regulation of platelet aggregation and thrombosis, modulation of glucose homeostasis, and influence on food intake [57]. The notable inhibition of *apoA-IV* expression following exposure to 2,7-DBCZ may affect lipid uptake and metabolism. However, lipid metabolism is inextricably associated with normal embryonic growth, and alterations in embryonic lipid metabolism can thus inhibit fetal growth. This is further supported by previous reports in which the addition of feed additives that improve lipid metabolism contributed to the early growth performances of embryos [58]. Therefore, it is reasonable to speculate that 2,7-DBCZ exposure alters the lipid metabolism pathway in *M. chulae*, thereby delaying the embryonic developmental processes. Previous studies have shown that 5-HMF causes cardiovascular developmental defects and increased pericardial edema by affecting the Wnt signaling pathway and cardiovascular development-related pathway [59]. Additionally, 2,7-DBCZ exposure can disrupt lipid uptake and metabolism in *X*. *tropicalis* embryos, resulting in developmental abnormalities in the cardiovascular system [37]. Exposure to the natural alkaloid berberine interferes with the homeostasis of angiogenic and cardiovascular developmental systems and induces pericardial edema in zebrafish in a time- and concentration-dependent manner [60]. Likewise, we suggest that 2,7-DBCZ treatment affects cardiovascular development-related pathways in *M. chulae*, thereby triggering pericardial edema.

## 5. Conclusions

In summary, our study revealed that 2,7-DBCZ exerts acute toxicity and developmental toxicity on *M. chulae* embryos, primarily manifesting as embryonic mortality and malformations such as pericardial edema and spinal curvature. Transcriptome analyses revealed that 2,7-DBCZ exposure primarily disrupts processes related to phototransduction, protein folding and processing, amino acid metabolism, lipid transport, and immune responses in biological systems. It also impacts the complement system and the metabolism of exogenous compounds, thereby inducing developmental toxicity in *M. chulae* embryos. By using the marine model organism *M. chulae*, our study provides valuable data on the toxicity of PHCZs in marine fish, thereby bridging gaps in our understanding of the impact of PHCZ pollutants in marine environments. Additionally, our study offers a valuable reference for the risk assessment and regulation of PHCZ contaminants in marine ecosystems.

## Figures and Tables

**Table 1:** Primer sequences of genes used to validate the RNA-Seq data.

Gene Name	Gene Name	Sequence	Gene Number
Reference gene	*β-* *A* *ctin*	F: GGCTACTCCTTCACCACCACAG	--
		R: TTCCGCAAGATTCCATACCG	
Up-regulated	*CYP1A*	F: TGCCACATTCAAGCCACATTACG	PB.21006
		R: ACAGTGGTATCGGGTATGGATTGC	
	*CYP1C*	F: CATCAGTGCTGCTTTGAGAGTGAC	PB.17128
		R: TTGGCTCGGAGACAGAAGTGAAC	
	*GST*	F: TTGCTTTCCTCGTCCGAATGGG	PB.1965
		R: GGGCGACTCTTTAGGTGGTTGTAG	
	*C3*	F: ATGCTCTTCTGGCTCTCGTCAAG	PB.7408
		R: CTCGGACACTGCCTGATACACC	
	*SQSTM1*	F: CCGCTCGTCCAGAGTTCAGTG	PB.8379
		R: ACGGCAGGCAGCAGAAGATTC	
	*IMPA*	F: TGCATCCTAACAGACGAACCTACC	PB.7771
		R:TAGACACTGCCACAAAGGGAATCC	
Down-regulated	*KRT1*	F: ACATGCGTCAGTCCGTAGAAGC	PB.4747
		R: GTCCTCCTCGTGGTTGGTCTTG	
	*RHO*	F: TGTAGGTCCCTCTCCATCTCTGTC	PB.13668
		R:GGTCCCTCTGTGCCGTTCATG	
	*KRT2*	F: GGTGGTGGTGGAAGCCGTATG	PB.18903
		R: GCACCTCCGCCTGATCTGAAG	
	*LEG1*	F: TTCTCTGGTGCTGCTGCTGTG	PB.12502
		R: ACATGATCGGACCGCCATTCTC	

**Table 2:** Summary of sequencing data statistics.

Sample	Raw Reads	Raw Bases	Clean Reads	Clean Bases	Error Rate (%)	Q20 (%) *	Q30 (%) *	GC Content (%)
2,7-DBCZ_1	44,405,146	6,705,177,046	44,178,282	6,605,033,687	0.0247	98.18	94.4	48.29
2,7-DBCZ_2	43,911,458	6,630,630,158	43,682,736	6,530,441,432	0.0246	98.24	94.58	48.28
2,7-DBCZ_3	46,485,840	7,019,361,840	46,222,348	6,922,762,351	0.0243	98.31	94.79	48.95
control_1	45,950,398	6,938,510,098	45,659,470	6,828,622,487	0.0248	98.13	94.33	49.47
control_2	51,691,138	7,805,361,838	51,434,326	7,679,874,890	0.0244	98.31	94.75	48.53
control_3	44,086,104	6,657,001,704	43,833,164	6,559,226,539	0.0244	98.3	94.77	48.71

*: Q20 and Q30 refer to the percentage of total bases with a sequencing quality of 99 and 99.9% or more, respectively. A Q20 value above 85% and a Q30 above 80% are generally considered acceptable.

**Table 3:** Statistical table of sequence alignment results.

Sample	Total Reads	Total Mapped	Multiple Mapped	Uniquely Mapped
2,7-DBCZ_1	44,178,282	36,539,927 (82.71%)	1,711,824 (3.87%)	34,828,103 (78.84%)
2,7-DBCZ_2	43,682,736	36,129,159 (82.71%)	1,600,659 (3.66%)	34,528,500 (79.04%)
2,7-DBCZ_3	46,222,348	38,521,041 (83.34%)	1,760,256 (3.81%)	36,760,785 (79.53%)
control_1	45,659,470	38,647,959 (84.64%)	1,947,722 (4.27%)	36,700,237 (80.38%)
control_2	51,434,326	42,871,351 (83.35%)	2,177,718 (4.23%)	40,693,633 (79.12%)
control_3	43,833,164	36,681,621 (83.68%)	1,884,808 (4.3%)	34,796,813 (79.38%)

## Data Availability

The data are contained within the article.
